# Association between CTLA-4 Gene Promoter (49 A/G) in Exon 1 Polymorphisms and Inflammatory Bowel Disease in the Tunisian Population

**DOI:** 10.4103/1319-3767.43285

**Published:** 2009-01

**Authors:** Walid Ben Alaya, Imen Sfar, Houda Aouadi, Saloua Jendoubi, Tawfik Najjar, Azza Filali, Yousr Gorgi, Taieb Ben Abdallah, Leila Mouelhi, Samira Matri, Khaled Ayed

**Affiliations:** Charles Nicolle Hospital, Immunology laboratory Ch. Nicolle Hospital, Tunis-1006, Tunis, Tunisia

**Keywords:** Crohn's disease, *CTLA-4*, gene polymorphism, inflammatory bowel disease, ulcerative colitis

## Abstract

**Background/Aim:**

To investigate the possible association between the polymorphism of the *CTLA-4* exon 1 +49 A/G and susceptibility to Crohn's disease (CD) and ulcerative colitis (UC) in the Tunisian population.

**Methods:**

The +49 A/G dimorphism was analyzed in 119 patients with CD, 65 patients with UC, and 100 controls by the polymerase chain reaction–restriction fragment length polymorphism method.

**Results:**

Significantly higher frequencies of the *CTLA-4* +49A allele and A/A homozygous individuals were observed in patients with CD when compared with controls (pc = 0.0023 and pc = 0.0003, respectively). Analysis of *CTLA-4* A/G polymorphism with respect to sex in CD showed a significant difference in A/A genotypes between female patients and controls (pc = 0.0001 and pc = 0.038, respectively). There were no differences in the subgroups of patients with CD.

**Conclusions:**

Forty-nine A alleles and AA genotype are associated with CD susceptibility in Tunisians. Other genes involved in the T-cell regulation remain strong candidates for IBD susceptibility and require further investigation.

Inflammatory bowel disease (IBD) is a chronic, inflammatory, intestinal disorder characterized by immune dysregulation and leukocyte recruitment into the gastrointestinal tract, which appears to be associated with microbial exposure.[1] Crohn's disease (CD) and ulcerative colitis (UC) are two distinct forms of IBD with some common clinical, epidemiological, and immunological features, but they can be distinguished by anatomical and histological features as well as by serologic markers.[2,3] Although the cause of IBD remains unknown, it has become evident that this disease is the result of abnormal immune responses induced by the interaction of environmental and genetic factors.[4] Genetic factors are known to play an important role in determining an individual's susceptibility to IBD. Significant linkages in chromosomes 1, 3, 6, 7, 12, 14, 16, and 19 have been reported.[5] However, *IBD-1*, a susceptibility locus in chromosome 16, was the first gene to be clearly associated with CD.[6,7] Some immunological perturbations seem to be shared by CD and UC patients, reflecting different pathways of the immune system-mediated chronic intestinal inflammation. CD is described as a T-helper (Th1) disease because the Th1 cytokines (IL-12, IFN-γ, and TNF-α) are the primary mediators of inflammation,[8] whereas UC is considered as an atypical Th2 disease according to mucosal cytokine profiles (IL-5, IL-4, and IL-13). However, this Th1/Th2 paradigm has recently been challenged by several authors.[8,9] Extensive lymphocyte proliferation has been shown with increased activated CD4+ and CD8+ T cells in the intestinal lamina propria of patients with UC and CD in several studies.[11–13] CTLA-4 is mainly expressed in activated T cells and plays an inhibitory role in regulating T-cell functions and self-tolerance.[14] Therefore, the molecules that mediate the T-cell regulation could influence disease susceptibility.[13] Cytotoxic T-lymphocyte antigen 4 (CTLA-4) is an inhibitory receptor expressed by activated T cells that seems to be an important downregulator of T-cell activation and might contribute to peripheral tolerance.[14] Thus, *CTLA-4* is a good candidate gene for susceptibility to IDB, because it acts as a negative regulator of T-cell activation and T/B, T/monocyte-macrophage cognate interaction. The *CTLA-4* gene is located on chromosome 2q33. At least three genetic polymorphisms have been reported in the human *CTLA-4* gene: one in the promoter region at position −318 consisting of a C/T transition,[15] a second in position +49 of exon 1 that lies in an A/G transition resulting in a threonine (Thr) or alanine (Ala) dimorphism,[16] and a third in the 3’ untranslated region with variable lengths of a dinucleotide (AT)n repeat.[17] Several studies have shown an association between an A/G single nucleotide polymorphism (SNP) in exon 1, position +49 of the *CTLA-4* gene, and autoimmune diseases such as type 1 diabetes,[18–20] multiple sclerosis,[21] Grave's disease,[22] HLA-DR4-rheumatoid arthritis,[23] and celiac disease.[24] However, studies on the polymorphisms of the *CTLA-4* gene in IBD have shown conflicting results in different populations.[25–27]

In this study, we have analyzed the *CTLA-4* exon 1 polymorphism (position 49 A/G ) in unrelated Tunisian patients with CD and UC to evaluate the contribution of the *CTLA-4* gene to genetic susceptibility to IBD.

## PATIENTS AND METHODS

### Patients

Blood samples were obtained from 119 patients with CD (62 male and 57 female) with a mean age of 34.65 years (range = 23−60 years), and 65 patients with UC (18 male and 47 female) with a mean age of 39.94 years (range = 25−74 years). A total of 100 healthy individuals (52 male and 48 female) matched for age (mean age = 32.4 years, range = 24−55 years), sex (40 male and 60 female), and ethnicity were included as controls. None of the healthy samples had any evidence of autoimmune diseases such as inflammatory bowel disease, diabetes, or other autoimmune diseases. All subjects were unrelated Tunisians treated at the Department of Gastroenterology of Charles Nicolle and La Rabta Hospitals in Tunis. The diagnoses of CD and UC were determined according to conventional endoscopic, radiological, and histological criteria.[28] Data obtained from each patient included age at diagnosis, disease location, behavior, and extraintestinal location, which were used to group the patients according to the Vienna classification.[29] None of them had any evidence of autoimmune diseases (type 1 diabetes, celiac disease, multiple sclerosis, Graves disease). All patients and controls gave informed consent to participate in this study that was approved by the Ethics Committee of Charles Nicolle Hospital in Tunis.

### Methods

Genomic DNA was extracted from peripheral blood leukocytes from all patients and controls using proteinase K and the phenol/chloroform method. Typing of the *CTLA-4* exon 1 A/G transition at position +49 was achieved by the polymerase chain reaction–restriction fragment length polymorphism method. The amplification reaction was carried out using primers: 5’CAAGGCTCAGCTGAACCTGGGT 3’ and 5’TACCTTTAACTTCTGGCTTTG 3’ and previously described cycling conditions.[19] The amplification products were digested with the restriction enzyme KpnI (Promega), and the fragments were separated on a 4% agarose gel. The A allele corresponds to the uncleaved fragment of 195pb.

### Statistical analysis:

Frequencies of the genotypes, alleles, and phenotypes were analyzed by using the χ^2^ test. The odds ratio (OR) and 95% confidence interval were calculated to measure the strength of the association observed. Hardy–Weinberg equilibrium was tested by calculating the χ^2^ for goodness of fit. Calculations were made by using Internet programs from www.myatt.demon.co.uk/epicalc.htm. Statistical significance was defined as *P* < 0.05. *P* values were corrected (pc) by Bonferroni correction for multiple comparisons, taking into account the number of alleles studied.

## RESULTS

Table 1 shows several clinical characteristics of 119 CD patients and 65 UC patients. We have genotyped 184 Tunisian IBD patients (119 CD, 65 UC) and 100 matched healthy controls for *CTLA-4* +49A/G polymorphism [Table 2]. All populations were in Hardy–Weinberg equilibrium. As the +49A/A genotype of *CTLA-4* was associated with IBD in the entire patient population, and as there was no significant difference in the +49A/G *CTLA-4* genotype frequencies in gender-grouped controls, we have compared the A/A genotype frequencies in various subgroups of IBD patients with those in the entire control population. When comparing CD patients with the control group, the frequency of the +49 A allele was found to be significantly higher in CD than in controls. Also, the distribution of the +49 A/A genotype was significantly higher in CD patients than in controls. On the other hand, when comparing UC patients with the control group, the frequencies of the +49A allele and the homozygous +49 A/A genotype were higher in UC patients than in controls, but those differences were not statistically significant when Bonferroni correction was applied. When we have analyzed the *CTLA-4* +49 A/G polymorphism with respect to gender [Table 3], we found that *CTLA-4* genotypes were similar in controls of both sexes (48 females and 52 males). However, the frequency of CD female patients carrying the +49 A/A genotype was significantly higher compared with that in the control group. The same result was obtained in women with UC when compared with that in the control group Thus, the homozygosity for the A allele could be a risk factor for development of IBD in female patients. A similar analysis has been done on male patients with CD and UC. The results showed a weak significant association in only CD patients compared with that in the control group. Subsequently, we sought to investigate whether this polymorphism could be linked to a particular clinical phenotype. While stratifying CD patients according to the Vienna classification, we were unable to find significant differences in allelic and genotypic +49 A/G *CTLA-4* frequencies compared with those in the control group. Likewise, subdivision of the UC patients according to the localization of the disease led to very small subgroups, too small to provide valid evidence for relevant observations. We did not find an association of +49 A/G polymorphism with the severity of disease in any of the IBD patients, as defined by the need for surgery (data not shown).

**Table 1:** Demographic characteristics and clinical features of Tunisian patients with Crohn's disease and ulcerative colitis

Crohn's disease	n (%)
Number (N)	119
Men	59 (49.5)
Women	60 (50.5)
Sex ratio	1.08
Age at onset	
Age < 40 years	91 (76.4)
Age > 40 years	28 (23.6)
Location	
Ileum	30 (25.2)
Colon	26 (21.8)
Ileocolon	63 (53)
Disease behavior	
Nonstricturing	
Nonpenetrating	66 (55.5)
Stricturing	38 (32)
Penetrating	15 (12.5)
Extraintestinal manifestations	
Ulcerative colitis	n (%)
Number (N)	65
Men	19 (29.2)
Women	46 (70.8)
Sex ratio	2.4
Age at onset	
Age < 40 years	28 (43)
Age > 40 years	37 (57)
Location	
Ulcerative proctitis	17 (26.1)
Left-sided	30 (46.1)
Pancolitis	18 (27.7)
Extraintestinal manifestations	
Arthritis	7 (10.7)
Dermatological	1 (1.06)
Arthritis	22 (18.5)
Dermatological	6 (5)

**Table 2:** Allelic and genotypic of +49 A/G CTLA-4 gene polymorphisms in patients with inflammatory bowel disease and controls

**+49 −Exon 1 A/G**	**Control**		**IBD**		**CD**		**UC**	
	(N = 100)		(N = 184)		N = 119)		(N = 65)	
				
	n	%	n	%	n	%	n	%
Genotype frequencies								
AA	8	8a	45	24b	33	27.7	c12	18.4d
AG	40	40	60	32.6	35	29.4	25	38.4
GG	52	52	79	42.9	51	42.8	28	43
Allele frequencies								
A	56	28e	150	40.7f	101	42.4g	49	37.6h
G	144	72	218	59.3	137	57.5	81	62.4

Comparing AA genotype frequencies in IBD patients with controls (ab): pc = 0.001 OR: 3.72, 95% CI, 1.60 < OR < 8.99

Comparing AA genotype frequencies in CD patients with controls (ac): pc = 0.0003 OR: 4.41, 95% CI, 1.82 < OR < 11.02

Comparing AA genotype frequencies in UC patients with controls (ad): pc = 0.077 OR: 2.60, 95% CI, 0.92 < OR < 7.52

Comparing A/Allele frequencies in IBD patients with controls (ef): pc = 0.003 OR: 1.77, 95% CI, 1.20 < OR < 2.61

Comparing A/Allele frequencies in CD patients with controls (eg): pc = 0.002 OR: 1.90, 95% CI, 1.24 < OR < 2.89

Comparing A/Allele frequencies in UC patients with controls (eh): pc = 0.065 Abbrevations: IBD: inflammatory Bowel Disease, UC: ulcerative colitis, CD: Crohn's disease

pc: *P* corrected, OR: odds ratio, CI: confidence interval.

**Table 3:** CTLA-4 exon 1 polymorphism analyzed with respect to sex in CD patients

	**Controls**		**CD females**		**CD males**	
	N = 100		N = 60		N = 59	
			
	n	%	n	%	n	%
Genotype frequencies						
AA	8	8	20	33.3	13	22
AG	40	40	13	21.6	22	37.2
GG	52	52	27	45	24	40.6
Allele frequencies						
A	56	28	53	44.1	48	40.6
G	144	72	67	55.8	70	59.3

CTLA-4 genotypes were similar in controls of both sexes: 52 males and 48 females; χ^2^ = 0.06, *P* = 0.8, two degrees of freedom

χ^2^ test of heterogeneity between CD females and controls (χ^2^ = 14.96 . 2 degrees of freedom, pc = 0.0001) odds ratio for AA genotype is 5.75 (2.17 < OR < 15.65, CI = 95%)

χ^2^ test of heterogeneity between CD males and controls (χ^2^ = 6.34, 2 degrees of freedom, pc = 0.022) odds ratio for AA genotype is 3.25 (1.15 < OR < 9.33, CI = 95%).

## DISCUSSION

The frequencies of the A allele and the AA genotype were significantly higher in patients with CD compared with those in the control group. Thus, we have demonstrated an association between a promoter polymorphism of the *CTLA-4* exon 1 A+49G and CD in this study. The significance of this association may differ according to the population studied and the type of inflammatory bowel disease, as also demonstrated for the *NOD2/CARD15* gene polymorphisms. Indeed, several studies have reported an association between CD and *NOD2/CARD15* mutation in Caucasians but not in UC patients.[7,30] In addition, evidence indicates that the type of inflammatory response occurring in the intestine of patients with CD differs from that in the UC patients. This probably reflects distinct pathways of immune activation and different types of cell recruitment in CD mucosa, where a Th1 response prevails with high IL-12 levels, whereas humoral immunity appears to be predominant in UC.[1,10,13] Stratification analyses revealed that the association was stronger in females than in males. Sex effects in IBD have been already reported by Fisher *et al*., who have identified several putative regions of sex-specific linkage. Regions on chromosomes 6, 11, 14, and 18 showed strong evidence of linkage in male-affected families but not in female-affected families.[31] Moreover, oral contraceptives have been shown to be associated with increased risk for CD.[32] Similar sex-specific linkage occurs in other immune-mediated diseases including type 1 diabetes, multiple sclerosis, and rheumatoid arthritis,[33,34] although the molecular basis for such effects is unknown. It has been proposed that epigenetic factors play an important role in the pathogenesis of IBD,[35] and that sex effects are mediated by sexual hormones, which have an effect on gene expression and consequently could lead to differential expression of disease susceptibility genes in males and females. According to our findings, it appears that *CTLA-4* gene polymorphisms increase susceptibility to CD in females in the Tunisian population. However, because there was no evidence of linkage of the *CTLA-4* gene with the X chromosome, it is possible that this difference could be a random variation. On the other hand, because there was no statistical difference in the A allele frequencies between male and female subjects, it is possible that the AA genotype could be a predisposing factor to CD in females. It is worthy of note that Yang *et al*. reported that females with allergic rhinitis had a significantly higher frequency of the A/A genotype in the *CTLA-4* +49 polymorphism than those without atopic diseases. On the other hand, males with and without allergic disorders exhibited no significant difference in *CTLA-4* +49 polymorphisms.[36] Our present results and the lack of previous studies on the sex distribution of *CTLA-4* polymorphisms in patients with IBD warrant further investigation. Previous studies assessed the prevalence of *CTLA-4* A+49G polymorphism in IBD patients with conflicting results.[25–27,37–39] Only two reports found an association of *CTLA-4* gene polymorphism with CD and/or UC. One study carried out on Japanese subjects with IBD found a high frequency of GG genotype at the A+49 G polymorphism in CD patients with fistula compared with those without it (*P* = 0.0388 ; OR = 2.67).[26] Another study carried out on Chinese patients with UC reported an association between the longer allele (122 bp) of the *CTLA-4* gene microsatellite polymorphism (AT)n and UC patients.[25] All other studies carried out on Caucasian British, Spanish, Dutch, Iranian,[27,37,39] or Chinese populations[27,38] did not find any association between *CTLA-4* gene polymorphisms and IBD. Not enough data are available to permit the conclusion that *CTLA-4* polymorphism is or is not associated with IBD. In this study, a strong association was found between the *CTLA-4* +49A allele and CD but not with UC, suggesting that the genetic factors for CD are distinct from those of autoimmune diseases. CD is not a true autoimmune disease, and has different associations at the *CTLA-4* exon 1 +49 G>A from all other autoimmune disorders. The reason for this discrepancy is not clear but might reflect an ethnic difference in the contribution of genetic factor(s). The association of several autoimmune diseases with the *CTLA-4* +49G allele has been explained as the result of a reduced inhibitory function associated with the G allele. Recently, Kouki *et al.* demonstrated that individuals homozygous for the G allele of +49 A/G have reduced control of T-cell proliferation compared with A/A homozygotes.[16] On the other hand, it has been shown that both the CTLA-4 protein and mRNA levels are significantly higher in individuals homozygous for the G allele.[40] Similarly, subjects that carry the G/G genotype have a greater T-cell proliferation response when suboptimally stimulated, compared with A/A homozygotes.[41] These findings might correlate with decreased negative regulation of T-cell proliferation, and therefore, predispose the individual to a greater risk of development of autoimmune diseases. The *CTLA-4* A/G polymorphism is unlikely to affect the function of the gene because 49 A/G is located on the peptide leader.[35] However, it is possible that the *CTLA-4* gene polymorphism affects CTLA-4 mRNA stability and subsequent CTLA-4 expression.[42] CTLA-4-deficient mice develop a lethal lymphoproliferative disease by massive, uncontrolled T-cell proliferation.[43] Although a study reported by Kouki *et al.* shows important evidence for functional differences among CTLA-4 variants, it is premature to conclude anything in the absence of data on all linked polymorphisms. *CTLA-4* has a significant but relatively small effect and does not seem to determine the phenotypic expression of CD. The chromosome 2q33 region contains also CD28 and ICOS, which are two other important immune regulatory genes that participate in regulating the T-lymphocyte-mediated immune response. Detailed analyses within the chromosomal region showed that linkage disequilibrium (LD) exists between *CD28*, *CTLA-4*, and *ICOS*, and a common haplotype is found very frequently among patients with autoimmune diseases and celiac disease.[44–46] As the existence of LD between *CTLA-4* and these two closely linked genes may mask the true causative risk allele for autoimmune disease, we cannot exclude that a single true disease-causing allele in *CTLA-4* or another neighboring gene might be found in this chromosome region. The role of *ICOS* in modulating T-cell function by coactivation, combined with its close physical proximity to *CTLA-4* and *CD28*, makes it an excellent candidate as a CD predisposition locus. Some polymorphisms have been detected in noncoding regions of the *ICOS* gene.[18] It is clear that further studies of this region are required to support or refute the possible location of a putative linked gene. Other possible candidates may be found among members of the *CFLAR-CASP10-CASP8* gene cluster that is positioned about 2.7 Mb distal to the *CD28-CTLA-4-ICOS* gene cluster.[47] This cluster encodes caspase 8, FADD-like apoptosis regulator (CFLAR), and caspase 10. Both caspases have been shown to be involved in CD95-mediated cell apoptosis. In addition, mutations in the *caspase 10* gene lead to the breakdown of lymphocyte homeostasis and the development of autoimmune lymphoproliferative syndrome.[48] As the apoptosis of activated T cells is an important mechanism of peripheral immune tolerance and a defect in apoptosis of mucosal T cells is an important contributing factor in the pathogenesis of IBD,[49] caspase-encoding genes are attractive candidates, but further studies are needed to pinpoint the causal polymorphism.

Our study had certain limitations: (i) we did not genotype all known *CTLA-4* SNPs but limited our study to the single polymorphism (*CTLA-4* exon 1 +49 A/G); (ii) it is possible that haplotypes exist in our population and we may have missed such haplotypes; therefore, more detailed linkage analysis of this chromosome region is required to identify the IDB susceptibility genes.

In this study, a *CTLA-4* exon 1 +49A polymorphism was associated with the development of CD but not UC, providing a strong support for an IBD susceptibility gene in the region surrounding *CTLA-4*. It remains to be determined precisely how the *CTLA-4* alleles influence the pathogenesis of IBD. Certainly, our results must await confirmation by other investigators. Because of the important role of CTLA-4 in the control of the inflammatory process and immune responses in IBD, our finding might, however, be compatible with the role of *CTLA-4* gene as a potential candidate gene.
